# Nonlocal Mechano-Optical Metasurfaces

**DOI:** 10.1021/acsphotonics.5c01385

**Published:** 2025-10-27

**Authors:** Freek van Gorp, Wenfeng Liu, Corentin Coulais, Jorik van de Groep

**Affiliations:** Institute of Physics, 84709 Universiteit van Amsterdam, 1098 XH Amsterdam, The Netherlands

**Keywords:** nonlocal metasurface, quasi-BIC, mechanical tuning, kirigami, multifunctional metamaterial

## Abstract

Tunable metasurfaces enable active and on-demand control over optical wavefronts through the reconfigurable scattering of resonant nanostructures. Here, we combine novel insights inspired by mechanical metamaterials with the unique sensitivity of nonlocal optical resonances to interparticle distances to achieve giant tunability in mechano-optical metasurfaces where the mechanical metamaterial and optical metasurfaces are integrated in a single nanopatterned material. In a first design, judiciously engineered cuts in a flexible substrate enable large, strain-induced extension of the interparticle spacing, tuning a high-quality-factor resonance in a silicon nanoparticle array across a very broad spectral range. In a second design, we eliminate the substrate and demonstrate a nanopatterned silicon membrane that simultaneously functions as a mechanical metamaterial and an optical metasurface with large tunability. Our results highlight a promising route toward active metasurfaces, with potential applications in tunable filters, reconfigurable lenses, and dynamic wavefront shaping.

## Graphical abstract

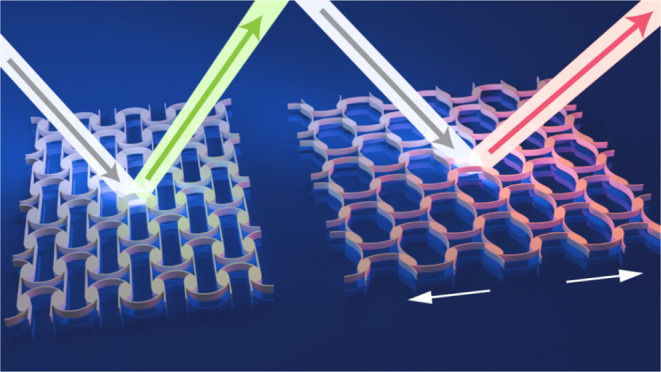

## Introduction

Nanophotonic metasurfaces employ engineered arrangements of interacting optical resonators to steer,^1^ filter,^2^ and shape optical wavefronts^3,4^ in ultracompact optical coatings. The optical function of such passive metasurfaces is imprinted in the nanoscale geometry and, therefore, challenging to manipulate postfabrication. Tunable metasurfaces enable active and on-demand control over optical wavefronts through the reconfigurable scattering of resonant nanostructures. This can be achieved by active manipulation of the optical properties of the resonant nanoparticles or its dielectric surrounding, for example using phase-change materials,^5–7^ thermal effects,^8,9^ electrostatic doping of metal-oxides,^10–13^ or electrochemical intercalation of electrochromic materials.^14^ Many of these approaches exhibit considerable potential; however, such tuning remains remarkably challenging in the visible spectral range, as the refractive index of photonic materials is inherently difficult to manipulate at optical frequencies.

A promising alternative approach is to employ mechanical manipulation of the nanoparticles, where the far-field interference of the locally scattered fields is effectively manipulated through changes in the interparticle spacing. Conventionally, such mechanical tuning of metasurfaces is achieved through relatively simple methods, such as stretching flexible substrates^15–20^ or employing out-of-plane deformations via MEMS technology.^21–23^ Although promising, metasurface tunability remains limited by the rudimentary mechanical manipulations of simple stretching and bending.

Recently, mechanical metamaterials have emerged as a novel method to control a material’s mechanical properties through their internal structure. Using the careful geometric design of compliant hinges and rotating elements,^24,25^ these metamaterials can combine low density with high strength and energy dissipation,^26,27^ perform computation,^28,29^ and exhibit advanced shape-morphing capabilities.^25,30–37^ Compared to simple stretching and bending, these mechanical metamaterials offer a distinct advantage for optical wavefront shaping by providing unique degrees of freedom through the internal rotations of their constituent building blocks, enabling more intricate and precise control over the system’s geometry.^38^ While initial demonstrations of light manipulation with metamaterial platforms have shown significant potential,^39–42^ and more recently at GHz frequencies using localized resonances combined with Pancharatnam–Berry phase control,^43^ these have been restricted to macroscale unit cells and operational frequencies in the GHz–THz regime, rely on resonant particles supported by mechanically limited substrates, and employ only localized resonances.

Here, we address these limitations by combining the unique sensitivity of nonlocal metasurfaces^44–46^ to interparticle spacing and orientation with the large internal rotations offered by flexible mechanical metamaterials^24,25,32,47–50^ to demonstrate a nanopatterned membrane that simultaneously functions as an optical metasurface and a mechanical metamaterial—a nonlocal mechano-optical metasurface. We exploit these large internal rotations to change the distances and angles between resonant nanoparticles on the fly, which in turn allows us to dynamically tune the resonance condition and associated optical filtering properties of the optical metasurface and to showcase the synergy between mechanical and optical functionalities in a single platform. Our approach augments the toolbox of reconfigurable metasurfaces and opens avenues for the synergistic design of mechanical and optical functionalities.

## Results

### Idealized Kirigami Metasurface

We first introduce a reconfigurable Metasurface made from resonant nanoparticles on a kirigami substrate that exhibits a tunable quasi-bound state in the continuum (q-BIC) with a high quality factor (*Q*) by simple stretching (Figure 1). Unlike conventional stretchable materials such as polydimethylsiloxane (PDMS),^51^ judiciously placed cuts in the kirigami substrate enable engineered mechanical deformations and thereby unusually large strain and internal rotations (Figure 1a–c).^52,53^

**Figure 1:**
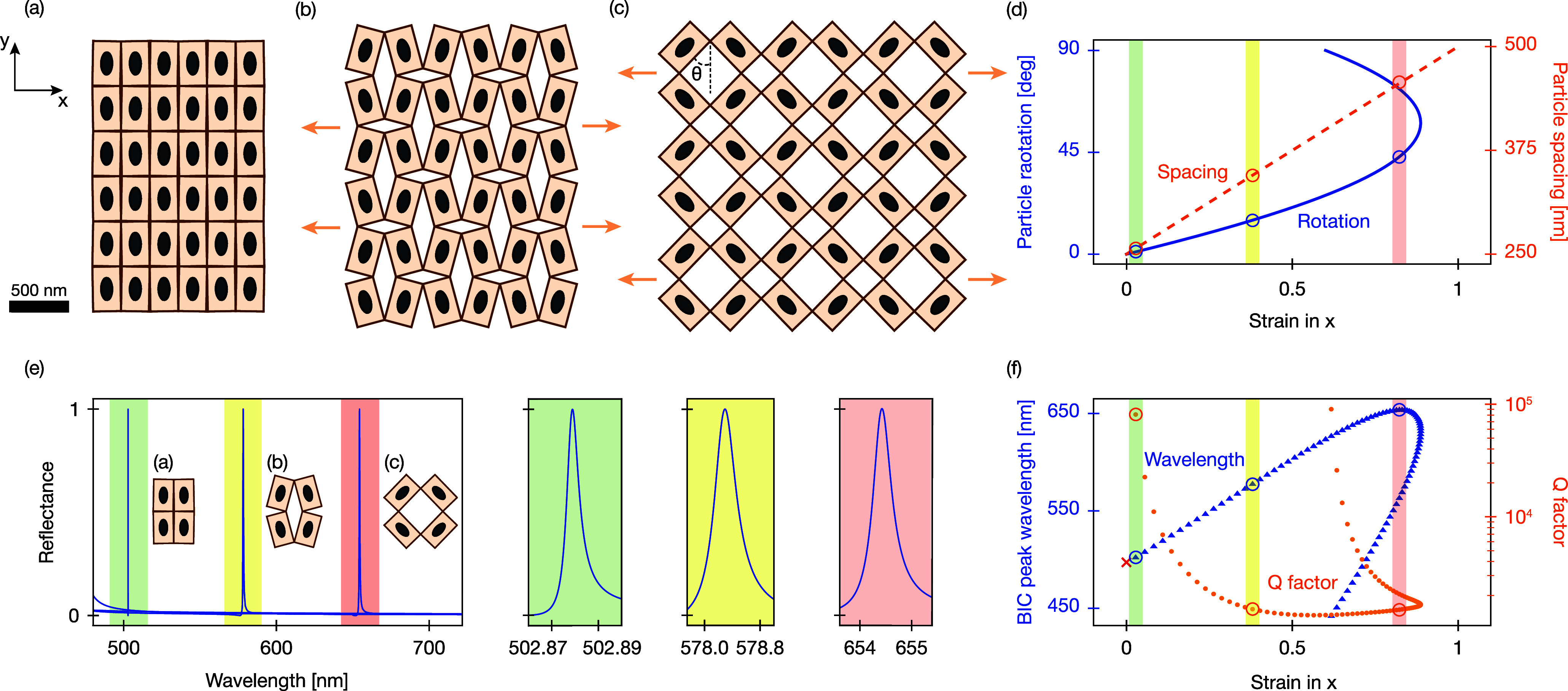
Mechanical deformation and tunable optical response of an idealized kirigami metasurface. (a–c) Schematics of 3 × 3 unit cell sections of the kirigami metasurface under increasing levels of strain: minimal strain (θ = 1°) (a), medium strain (θ = 15°) (b), and large strain (θ = 44°) (c). Each elliptical nanoparticle is centered on a rectangular tile measuring 250 × 400 nm. (d) With increasing strain in the *x*-direction, the tiles—and thus the particles—are rotated by an angle θ (solid, blue) and displaced, leading to changes in the interparticle spacing (dashed, orange). (e) Reflectance spectra for the arrays shown in (a–c), illustrating the giant tunability of the high-quality-factor resonant peak across the visible spectrum from green (∼500 nm) through yellow (∼580 nm) to red (∼650 nm), with zoomed-in sections near these wavelengths to highlight the Fano line shape. (f) Strain dependence of the resonance wavelength (blue triangles) and quality factor *Q* (orange circles).

The kirigami is decorated by an array of nanophotonic resonators^54,55^ —each elliptical resonator is placed in the center of each rectangular tile. Crucially, the periodicity of the Metasurface and the kirigami substrate must be commensurate. When the kirigami is strained along its *x*-axis, the angle θ (Figure 1c) varies from 0 to ∼58°. Importantly, the interparticle distance (pitch) also increases linearly with applied strain, reaching up to 89% elongation (Figure 1d). As such, a wide range of relative spacings and orientations between the resonators can be obtained on demand by simply stretching the kirigami Metasurface. In the corresponding optical simulations, we treat the substrate as fictitious (i.e., *n* = 1) and use a constant, lossless refractive index for silicon.

### Giant Tunability

To test whether stretching the kirigami leads to notable changes in the optical response, we use finite-element frequency-domain simulations with a normally incident *x*-polarized plane wave to calculate the reflection spectrum for varying metasurface geometries (see the Methods in SI). The reflection spectrum of the kirigami metasurface (Figure 1e) reveals a single resonant peak with three remarkable features. First, as the kirigami metasurface is stretched, the spectral position is tuned across the spectrum from 500 to 650 nm (Figure 1f) due to the strong dependence of the q-BIC mode on the interparticle spacing. This range corresponds to 328× the resonant line width. This giant tuning range is in stark contrast to the relatively small tunability of most existing platforms for active metasurfaces.

Second, the resonance quality factor decreases from theoretically arbitrarily large for infinitesimally small deformation to a minimum of *Q* = 1334 at θ = 24° (Figure 1f). Interestingly, this decrease is less pronounced than the *Q* ∝ sin^–2^θ that is characteristic of the perturbation of q-BIC modal symmetries.^55^ We attribute this to the changes in particle spacing associated with the particle rotation within the kirigami metasurface (See SI). Third, the maximum spectral shift is achieved for θ_max_ = 44°, which shows an unexpected discrepancy with the 58° that provides the largest displacement based on the kirigami design.

### Quasi-Bound State in the Continuum

To elucidate the interplay between internal rotations and nonlocal resonances, we present in Figure 2 the electric field lines (arrows) and the intensity enhancement (color) at resonance for three levels of strain (ε; the unstretched metasurface (ε = 0, Figure 2a), moderate strain (ε = 0.38 Figure 2b), and large strain (ε = 0.83 Figure 2c). The individual ellipsoidal particles support a strong electric dipolar Mie resonance, with the dipole moment (anti)­parallel to the major axis. However, for the unstretched situation, the modal symmetry of the unit cell inhibits incident light from coupling to this pure BIC, and the dipolar field lines are not observable (Figure 2a). The resulting field intensity in the plane of the metasurface is not significantly enhanced beyond the incident intensity: |*E*|^2^ < 10 |*E*_0_|^2^. For nonzero stretch ratios, the unit cell expands, the particles rotate, and the modal symmetry of the unit cell is perturbed. For small angles (Figure 2b), the electric field lines of the dipolar Mie modes are clearly observable in the individual particles, with strong field intensities concentrated at the particle tips. Careful evaluation of the field lines shows that within a four-particle unit cell, neighboring dipole moments (white arrows guide the eye) are oriented antiparallel with a relative angle close to θ. Resonant coupling to this q-BIC mode gives rise to strong field enhancements in the plane of the metasurface (|*E*|^2^ ≫ |*E*_0_|^2^). Interestingly, for large strain, the effective dipole orientation within the individual resonant nanoparticles is no longer aligned with the long axis of the ellipsoidal particle, but is reoriented toward smaller θ (Figure 2c). This intuitively explains why the maximum spectral shift does not align with the maximum mechanical displacement. For θ = 90°, the structure returns to a symmetry-protected BIC where the dipoles align with the *y*-axis again (Figure S4), albeit at a lower resonance wavelength than the original BIC (Figure 1f).

**Figure 2:**
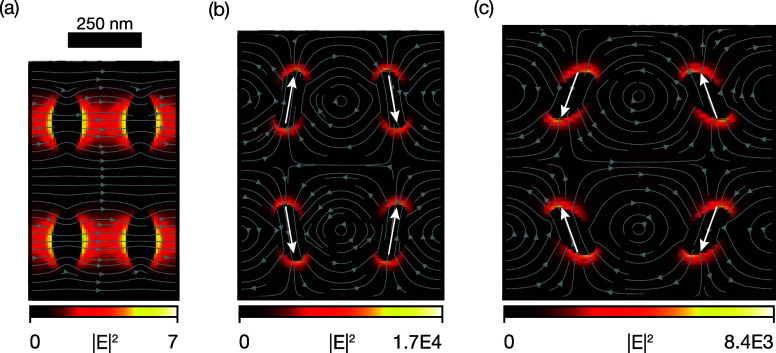
Electric field intensity profiles, normalized to the source intensity, for the idealized kirigami metasurface. Gray (arrow) lines indicate the electric field lines in the plane. Overlaid white arrows indicate the effective dipole moments. (a) The undeformed state (θ = 0°) displays a negligible field enhancement. (b) Small strain condition (θ = 15°) and (c) large strain condition (θ = 44°) show enhanced localized field intensities.

Further evidence for the collective, nonlocal nature of this mode is provided in the Supporting Information, where we show that the resonance wavelength follows a parabolic dispersion as a function of *k*_∥_ (Figure S6) and that 1/*Q* scales with (*k*_∥_/*k*_0_)^2^ (Figure S7), consistent with the behavior expected for a q-BIC.

### Beam-Linked Metasurface

So far, we have explored the mechano-optical response of an “ideal” kirigami metasurface where the substrate was fictitious (i.e., *n* = 1) and the refractive index of the silicon was constant and lossless. In the following, we aim to address these physical limitations in a second design: we include the dispersive and lossy optical constants of realistic silicon, and the particles are mechanically supported. Crucially, our design is composed of a nanopatterned 50 nm-thick silicon membrane that simultaneously functions as a mechanical metamaterial and as an optical metasurface (Figure 3a–c). To this end, the optically resonant nanoparticles are connected by carefully designed 10 nm wide beams. We optimize the shape of the beams such that (i) they bend to emulate the counter-rotation mechanism of the rotating rectangle as closely as possible (see SI for a detailed discussion); (ii) the stress remains below the failure limit when the metasurface is stretched to a maximum of 60° (Figure 3e). This is enabled by the notable enhancement of the strength of silicon at the nanoscale.^56^ In practice, the attainable fracture strength of single-crystal silicon nanobeams depends on the morphology. In particular, sidewall surface roughness reduces the nominal fracture strength from 20 GPa to values in the 12–16 GPa range.^57,58^ These values still exceed the maximum stresses obtained in our simulations (<10 GPa at θ = 60°), and further reduction of the operational strain would bring stresses well below even the conservative strength estimates. (iii) they minimally affect the optical resonance of the nanoparticles.

**Figure 3:**
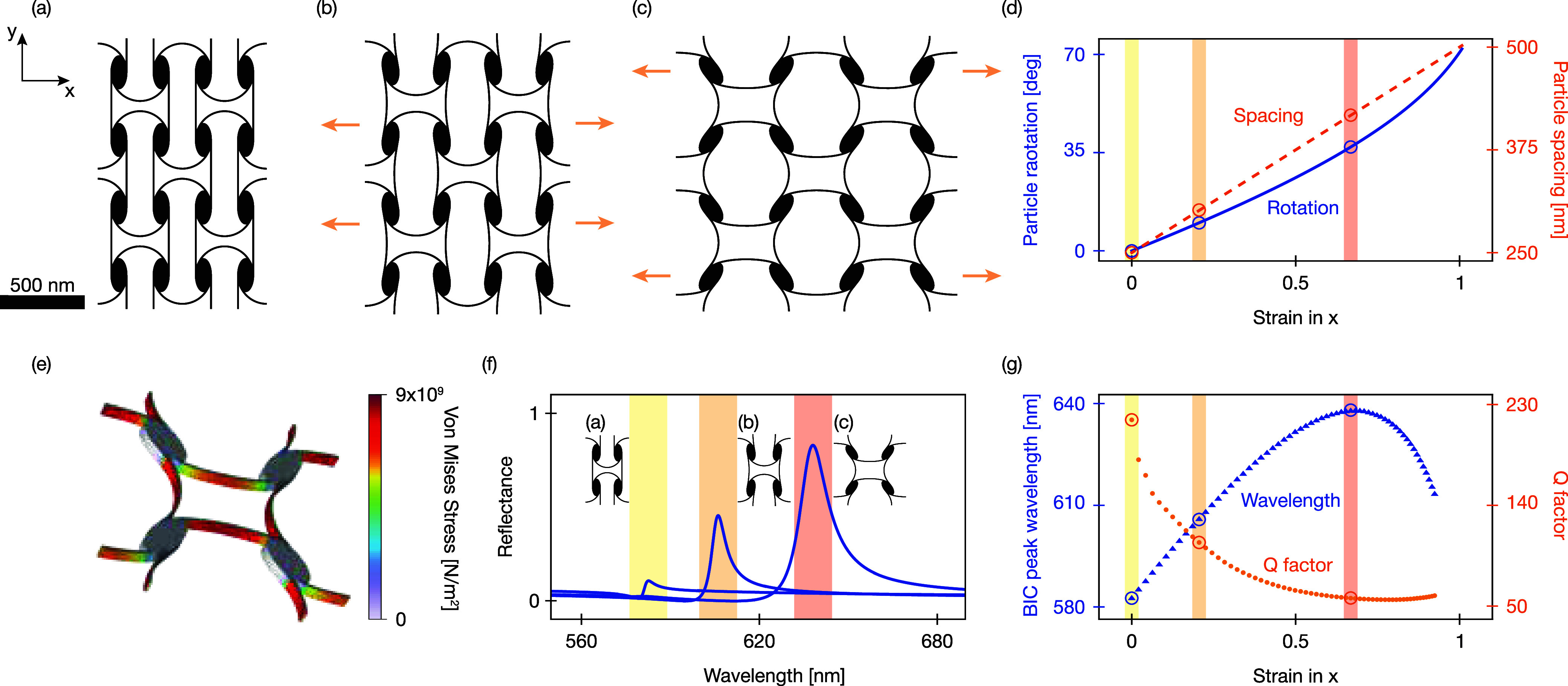
Mechanical deformation and tunable optical response of the beam-linked metasurface (a–c) Schematics of 2 × 2 unit cell sections of the kirigami-inspired metasurface: the initial undeformed state (a) shows no rotation of the elliptical nanoparticles, while moderate strain (θ = 10°) (b), and large strain (θ = 38°) (c) show increasing rotation and interparticle spacing. (d) Particle rotation angle θ (solid, blue) and displacement (dashed, orange) as functions of strain in the *x*-direction. (e) Simulated stress distribution for θ = 60°, showing strong localization of the stress at the beams. (f) Reflectance spectra for the arrays shown in parts (a–c), illustrating the large tunability of the resonant peak across the visible spectrum from yellow (∼580 nm) through orange (∼610 nm) to red (∼640 nm). (g) Strain-dependence of the resonance wavelength (blue triangles) and quality factor *Q* (orange circles), with a notable overall decrease in comparison to Figure 1f.

Analogous to the ideal design (Figure 1), applying in-plane strain initiates a predesigned mechanical deformation of the particle’s angle and interparticle spacing (Figure 3d). Just as for the idealized kirigami metasurface, the beam-linked metasurface’s interparticle spacing is directly proportional to the system strain. The particle rotation, however, is slightly increased over the relevant strain range in the beam-linked version. To assess the mechanical stability of the design, we perform simulations of the structural deformation and map the maximum stress throughout the unit cell (Figure 3e), confirming the mechanical stability under strain.

The introduction of optical damping and constrained mechanical deformations impacts the optical response in four distinct manners: (i) The spectral tunability of the resonance is reduced to 580–640 nm (Figure 3f,g). Despite being notably smaller than for the idealized kirigami metasurface, this spectral shift still corresponds to 4.7 times the largest resonance line width. (ii) The peak reflection is now limited by optical absorption and thus no longer reaches unity. (iii) The associated resonance quality factor is reduced from *Q* = 1336–*∞* to *Q* = 55–215 (Figure 3g). Although this may seem as an undesired effect, a finite resonance bandwidth is a prerequisite, e.g., reflective displays that employ structural color to provide sufficient optical power within the reflection peak. (iv) The introduction of the beams perturbs the unit cell symmetry and already enables weak coupling to the q-BIC mode for θ = 0°, i.e., the mode is no longer bound (Figure 3f). This symmetry breaking also occurs for very small initial rotations or due to imperfections in the array, and therefore does not significantly affect the tunability. At the same time, this symmetry breaking by the connecting beams is unavoidable, as the design uses the internal rotations of the unit cell to enable larger strain with reduced stress.

To further assess the impact of the nanobeams on the optical response, Figure 4 shows the field profiles on resonance for θ = 0° (a), θ = 30° (b), and θ = 60° (c). Indeed, despite the absence of particle rotation for θ = 0° (Figure 4a), the field intensity is >200, the incident field as a result of the formation of nanoscale hot spots at the contact points with the beams. Also, for nonzero θ, the contact points of the beams with the particles form sharp corners that perturb the modal field profile and give rise to strongly localized hot spots due to the required continuity of the displacement field *D* = ϵ*E*.

**Figure 4:**
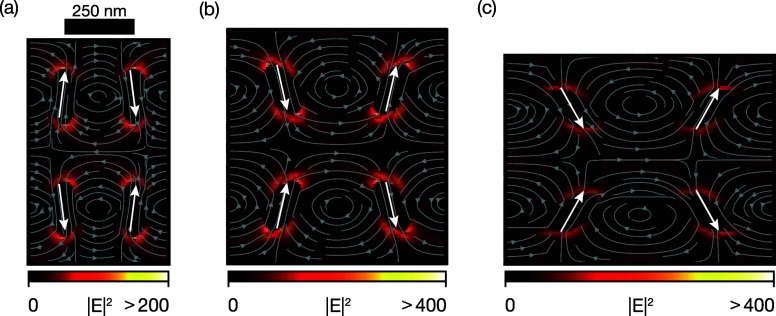
Electric field intensity (|*E*|^2^) profiles, normalized to the source intensity, for the beam-linked metasurface. Gray (arrow) lines indicate the electric field lines in the plane. Overlaid white arrows indicate the effective dipole moments. (a) The undeformed state (θ = 0°) already displays significant field enhancement. (b) Moderate strain condition (θ = 30°) and (c) high strain condition (θ = 60°) also show field enhancement.

## Discussion

### Finite-Size Effects

Boundary conditions are also known to play a crucial role in mechanical metamaterials and, in particular, they are known to affect how internal rotations permeate into the bulk.^48^ Our beam-linked metasurface does not escape this rule, and indeed we observe bulging at the edges, as a result of the nonslip boundary conditions (Figure 5a,b). This leads to a nonhomogeneous interparticle distance throughout the material, where the unit cells close to the edges do not stretch as much as the bulk of the metasurface (Figure 5b). The spatial extent of this inhomogeneous deformation is further quantified by a characteristic length scale $l^{*}=1500$ nm (3 unit cells) (Figure 5c), extracted from cross sections of the local strain throughout the 20 unit cells. The inhomogeneous mechanical deformation impacts the overall optical response of the metasurface. Averaged over the full metasurface, the reflection peak is slightly broader and exhibits a reduced amplitude (Figure 5d). However, avoiding only the regions within a distance $l^{*}$ from the mechanical clamps will already retain the optical response of the bulk material (Figure 5d). As such, the finite-size effects within the metasurface become negligible for all practical purposes.

**Figure 5:**
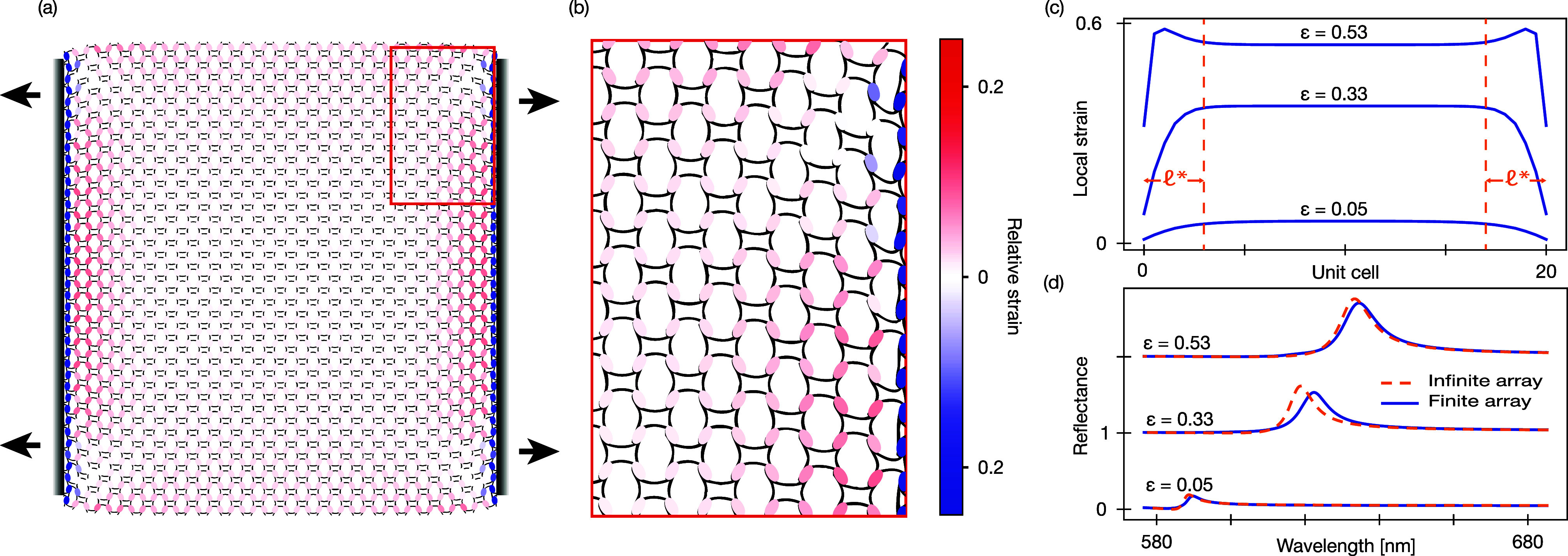
Finite-size effects of the realistic kirigami metasurface. (a) A finite-size metasurface (20 × 20 unit cells) with rigid mechanical handholds on either side, showing variations in local deformation. The color of the particles indicates deviation in the local strain with respect to the bulk strain. (b) Zoomed-in view of the local strain distribution near the handholds, highlighting localized deformation. (c) Local strain distributions across the structure for three strain values, each showing a strain plateau with roughly equal lateral size (between vertical dashed lines). (d) Comparison of the reflectance spectra between the finite array (solid blue curves) and an infinite array (dashed orange curves), illustrating how nonuniform stretching affects the overall optical properties, resulting in a reflectance peak with reduced amplitude and larger line width for the finite array.

## Conclusion and Outlook

In summary, we demonstrate how mechanical deformations in mechano-optical metasurfaces can tune a nonlocal q-BIC across the visible spectrum. The nanopatterned silicon membrane proposed here combines the function of a mechanical metamaterial and optical metasurfaces in a single structure and alleviates the need for a supporting substrate.

Beyond resonance peak shifts that are characteristic of elastomer-mounted/embedded photonic structures, our approach leverages internal rotations to dynamically reorient as well as reposition scattering particles. This additional degree of freedom enables not only resonance wavelength tuning but also active control over the resonance quality factor, providing a controllable coupling between *Q* and λ that is inaccessible to simple stretching substrates. This highlights how mechanical design can enrich the optical response and control of metasurfaces. Moreover, this concept opens up opportunities for multistable metasurfaces, mode switching with possible sign changes in dispersion, switchable lensing, beam steering, and polarization control, pointing to a broad class of functionalities beyond reflective filtering.

Our results pave the way for controlling light fields using designer mechanics with the prospect of more complex mechanical deformations and tunable optical functions for dynamic beam steering and wavefront manipulation beyond reflective filtering. Looking forward, the experimental demonstration of this concept requires high nanofabrication precision, typically enabled by electron-beam lithography (EBL), combined with novel in-plane mechanical actuation mechanisms that ideally capitalize on established MEMS technology. While EBL is not scalable, substrate conformal imprint lithography (SCIL) offers a scalable alternative with a demonstrated 6 nm resolution.^59^ This approach opens up new opportunities for translating advanced metasurface concepts into practical large-scale applications.

## Supplementary Material



## Data Availability

All data and analysis scripts underlying the figures presented in this work are freely available in the form of a replication package at 10.6084/m9.figshare.30350479.

## Notes

Detailed simulation methods; BIC quality factor scaling details; Design of the beam-linked metasurface; Poisson’s ratio of the kirigami substrate; Y-polarization BIC and dipole moment alignment with minor axis; q-BIC dispersion; Actuation (PDF)
