# Supplementary material for: Nonlocal Mechano-Optical Metasurfaces
Source: ACS Photonics. 2025 Oct 27;12(11):5978–84. doi: 10.1021/acsphotonics.5c01385 (PMC12637858; doi:10.1021/acsphotonics.5c01385)
Supplement: Supplementary file 1 [file ph5c01385_si_001.pdf]

Nonlocal mechano-optical metasurfaces: Supporting information

Freek van Gorp, Wenfeng Liu, Corentin Coulais, and Jorik van de Groep*

Institute of Physics, Universiteit van Amsterdam, 1098 XH Amsterdam, The Netherlands

E-mail: j.vandegroep@uva.nl

Methods

To test whether stretching the kirigami leads to notable changes in optical response, we use commercially available finite-element method software (COMSOL Multiphysics version 6.0).[1] The simulations for the idealized metasurface use an iterative solver (GMRES) in the RF module. The metasurface has ellipsoidal nanoparticles with a height of 50 nm, semimajor axis and semiminor axis lengths of 100 nm and 50 nm, respectively. The position of the particles follows the motion of the rotating rectangles kirigami mechanism. Assuming infinitesimally sized hinges this is trivial to determine analytically. The nanoparticles are assumed to have a constant refractive index of 3.47 and are surrounded by air with a refractive index of 1 (i.e. there is no physical substrate). Both the particles and the surrounding air are considered to be completely lossless. The simulation domain consists of a single nanoparticle with perfect electric conductors and perfect magnetic conductors to mirror the fields in the x- and y-directions. This approach achieves periodic boundary conditions at a quarter of the computational cost compared to conventional periodic boundary conditions. Note that this assumes the incident wave, metasurface, and all outgoing waves all share these mirror symmetries. Port boundary conditions were applied in the z-direction, 900 nm away from

the metasurface. The metasurface is illuminated under normal incidence with a plane wave ($\lambda = 400 - 750$) nm, with the electric field polarized along the x-axis. The simulation domain is divided into three distinct regions: the silicon nanoparticles, the surrounding area immediately adjacent to the particles, and the air regions further above and below the metasurface. Meshing for all regions is generated automatically using COMSOL's *General Physics* meshing algorithm, with element sizes tailored to each region for accuracy and computational efficiency.

For the beam-linked metasurface the optical response is also calculated using COMSOL Multiphysics version 6.0. However, the beam-linked metasurface diffracts light within the spectral region of interest. As a result, the simulations require periodic boundary conditions, instead of the 'mirror' boundary conditions. The simulation domain therefore includes the full four-particle unit cell. With these changes COMSOL's suggested solver changes to a direct solver (PARDISO). The ellipsoidal nanoparticles retain the same dimensions as in the previous simulation. However, they are now interconnected with silicon beams and we now account for dispersion and losses using material properties based on the silicon refractive index data from Schinke et al. (2015).[2] The meshing for the optical simulations is adjusted to account for the added complexity of the beam-linked geometry. In particular, the *maximum element growth rate* and *curvature factor* were refined to accurately resolve the connections between the ellipses and beams. Regions near the silicon beams are meshed more finely to capture the strong field gradients and mechanical deformation. Mechanical deformation of the beam-linked metasurface is simulated using the PARDISO direct solver in COMSOL's solid mechanics module. A single unit cell is constrained at the connecting beams to prevent displacement along the y- and z-axes, while a step-by-step displacement is applied in the x-direction to mimic stretching. The resulting deformed mesh, at strain values of interest, is then exported for use in optical simulations.

Finally, for the mechanical deformation for finite-sized beam-linked metasurfaces we use the "standard" solver in the commercial software Abaqus (2020)[3] for the finite-element sim-

ulation. We model the kirigami structure of 20×20 unit cells with a linear elastic material model. The model uses plane stress conditions with quadrilateral elements (CPS4R). We construct the mesh so that all of the ligaments in the unit cells are two elements across. The left and right boundaries of the kirigami are attached to a rigid plate and only the right plate is allowed to move in the stretching direction. The other two sides of the kirigami are free to rotate. We perform static stretching analysis on the model with the displacement control.

Quality factor scaling

In *Asymmetric Metasurfaces with High-Q Resonances Governed by Bound States in the Continuum* Koshelev et al.[4] show the (radiative) quality factor scales with an asymmetry parameter α as:

$$Q_{rad} \propto \alpha^{-2}. \tag{S1}$$

For the square lattice of tilted silicon-bar pairs, as described in their article, the asymmetry parameter $\alpha = \sin(\theta)$.

The ideal kirigami metasurface is similar to their square lattice, with two distinct changes. Firstly, the unit cell goes from two to four particles as each neighbouring particle rotates in the opposite direction, rather than each entire column rotating in the same way. Secondly, the particles don't just rotate, the spacing between the particle also changes. To show that the quality factor scaling differs from Koshelev's prediction only because of the displacement, we also simulate the four-particle unit cell without displacements (Fig. S1). The quality factor scaling of the 'non-displacing metasurface' perfectly follows the prediction, even for relatively large angles, while the ideal kirigami metasurface only approaches the predicted scaling for $\theta < 1°$.

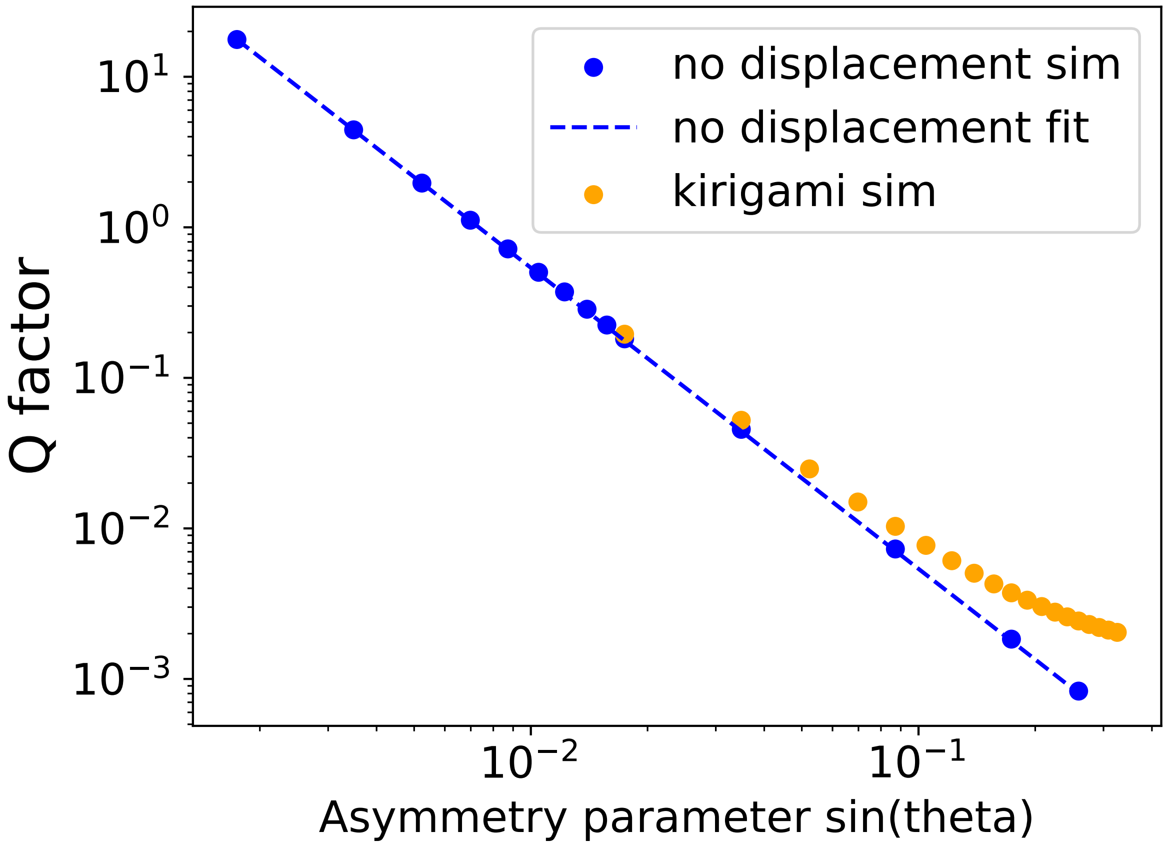

Figure S1: **Quality factor (normalized by unit cell area) scaling with asymmetry parameter α.** For the metasurface without changing inter-particle spacing (blue circles/dashed line). For the metasurface with the full mechanical motion (orange circles).

Design of beam-linked metasurface

The mechanical design of the beam-linked metasurface is inspired by the 'rotating rectangles' kirigami mechanism. This design is known for allowing large strain through internal rotations. Both the large strain and internal rotations are desirable deformations for in our optical metasurface design; the strain enables wavelength shifts and the rotation allows tuning of the quality factor.

While the design inherits the counter-rotational dynamics of the original kirigami tessellations, a significant modification is introduced in the placement of the connected beams relative to the nanoparticles. In an initial design, the shorter beams connecting the nanopar-

ticles were not pre-curved. The short beams experience large stress even at relatively small strain because of increased particle rotation at reduced strain values (Fig. S2). Once the particles reach a certain angle, their further rotation no longer enables larger strain. This in turn causes the beams to elongate under high stress. By simply introducing initial curvature to the beams, the strain causes the beams to straighten rather than elongate under stress.

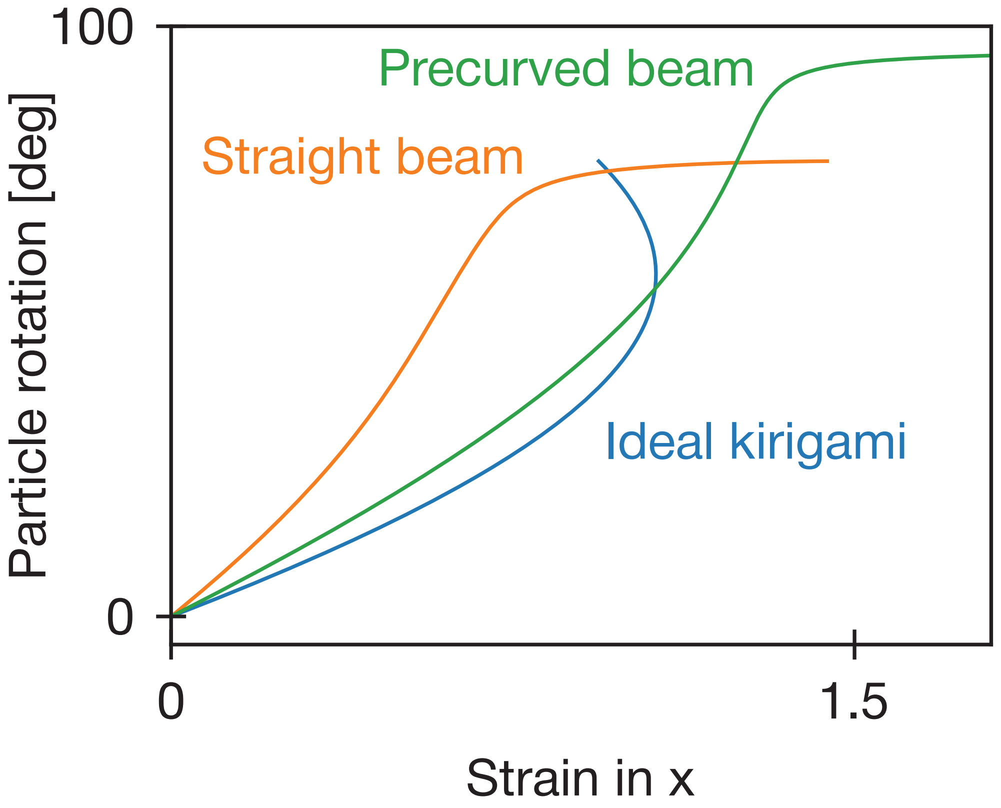

Figure S2: **Particle rotation as a function of strain in kirigami metasurface designs.** The ideal kirigami metasurface employing the 'rotating rectangle' mechanism (blue) is characterized analytically. The naive beam-linked metasurface, constructed with only straight beams (orange), exhibits a plateau, indicating significant stress due to beam elongation. The optimized beam-linked metasurface (green) shows a similar plateau but at substantially higher strain values, reflecting improved performance.

Poisson's ratio

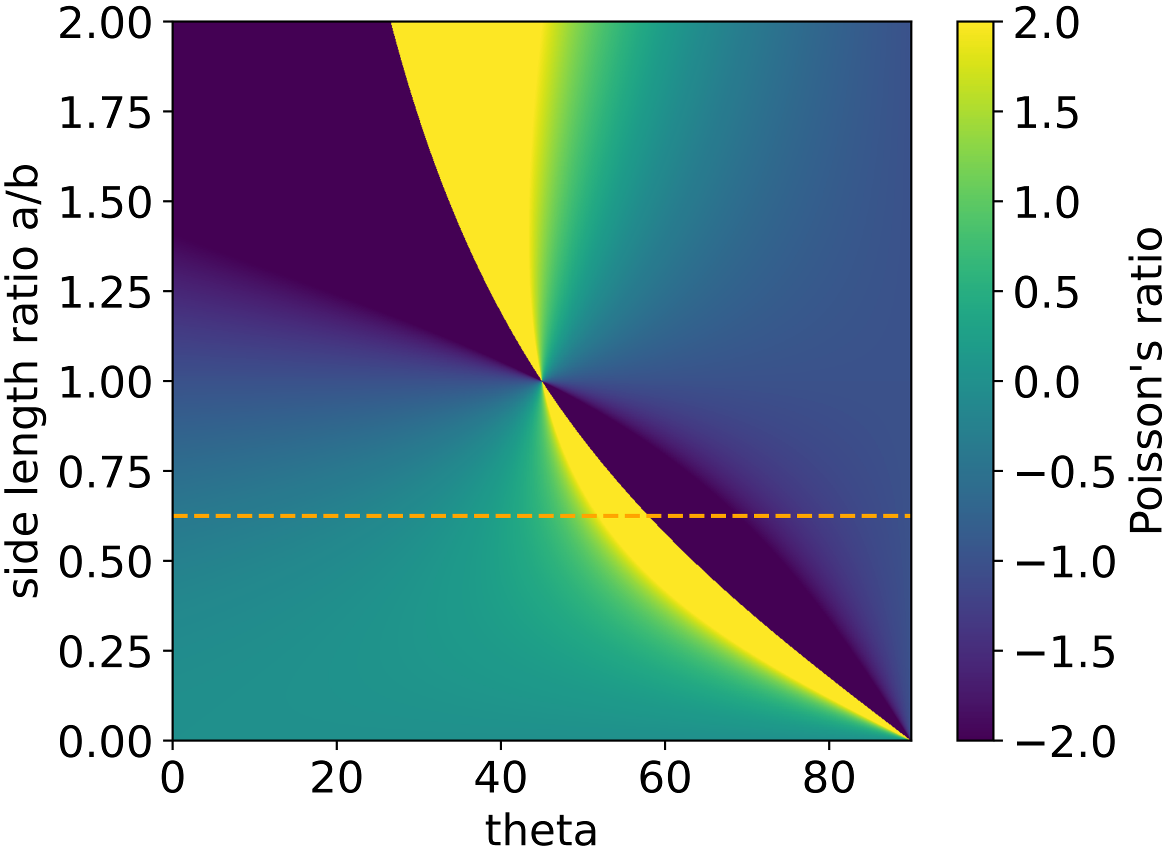

Figure S3: **Poisson's ratio for kirigami 'rotating rectangles' mechanism.** The ratio of the rectangle side lengths fully determines the behavior of the kirigami when subjected to strain. The dashed orange line is the ratio used for the metasurfaces.

The strain for the idealized metasurface is the analytical strain of the rotating rectangles. To determine that we first calculate the length in the x-direction L for any given angle θ. It is trivial to see the length is given by

$$L = a\cos(\theta) + b\sin(\theta), \tag{S2}$$

where θ is the tile rotation angle, and a (250 nm) and b (400 nm) are the rectangle side

lengths, with a the length in the strain direction. The strain ε is simply

$$\varepsilon = \frac{L - L_0}{L_0}, \tag{S3}$$

where $L_0 = L(\theta = 0) = a$. In terms of the angle θ this can be rewritten to:

$$\varepsilon = \frac{a\cos(\theta) + b\sin(\theta) - a}{a}. \tag{S4}$$

The angle for which this strain is maximized is:

$$\theta_{max} = \operatorname{atan}\left(\frac{b}{a}\right). \tag{S5}$$

There is, of course, also strain in the y-direction as a result of the elongation in the x-direction. The ratio of these two strains is known as Poisson's ratio. Normal materials have a Poisson's ratio greater than zero. However, auxetic materials have a negative Poisson's ratio, meaning if they are uniaxially stretched(compressed) they get wider(narrower) in the other direction; the opposite of what we expect from normal materials. Our metasurface designs exhibit auxetic behavior. In fact, the Poisson's ratio of the rotating rectangles is a function of the strain:

$$\nu_{ab} = -\frac{d\varepsilon_y}{d\varepsilon_x} = -\frac{-\sin(\theta) + \frac{a}{b}\cos(\theta)}{-\sin(\theta) + \frac{b}{a}\cos(\theta)}. \tag{S6}$$

This ratio starts out negative and becomes positive for all values of a and b where a≠b. For the rotating square case, $a = b$, the Poisson's ratio is a constant -1. For rectangles, this means while straining the material it first exhibits auxetic behavior, then non-auxetic behavior before reaching its maximum strain (Fig. S3).

Straining a substrate cut in the rotating rectangles pattern will therefore initially increase the inter-particle spacing in both directions, and while continuously increasing the distance between the particles in the stretching direction, start decreasing it in the other. Therefore, the lengths of the substrate unit cell is an interesting design parameter for changing the

inter-particle spacings under strain.

Y-polarization BIC

In the main text we show the idealized metasurface's BIC looks like dipole moments oriented along the individual ellipsoidal particle's major axes. For larger rotations, and thus greater symmetry breaking, the dipole moment misaligns with the major axis. In Fig. S4 we see that for large angles the dipole moment starts to align with the minor axis of the particles instead. In fact, at exactly 90° the mode is once again forbidden by symmetry; a second BIC.

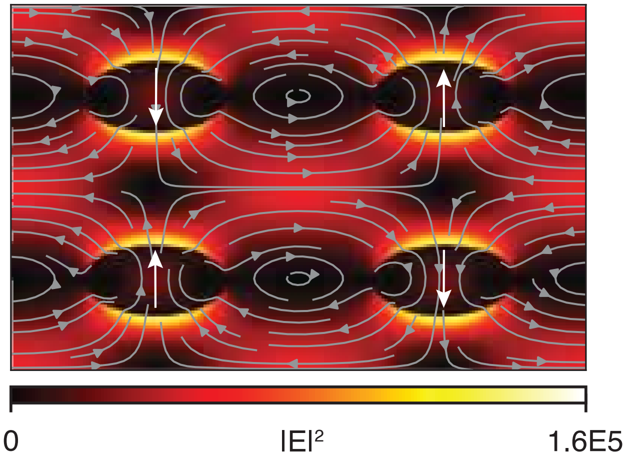

Figure S4: **Electric field intensity profile, normalized to the source intensity, for the idealized kirigami metasurface at $\theta = 89°$ for a normal incident x-polarized field.** Gray (arrow) lines indicate the electric field lines in the plane. Overlaid white arrows indicate the effective dipole moments, showing they align with the ellipsoidal particle minor axis.

In the main text we have only shown the qBIC for x-polarized incident plane waves. However, this same qBIC exists for y-polarization. For the idealized kirigami metasurface we simply rotate everything by 90° - including the strain axis - to see it really is exactly the same. ie. The metasurface for x-polarized light for particle rotation θ is equivalent to the metasurface for y-polarized light for particle rotation $90 - \theta$.

For the beam-linked metasurface, this mapping no longer holds. The pre-curved beams changes the strain behavior in the different directions, so the qBIC does not replicate the x-polarized trend under a 90° shift. As shown in Fig.S S5, the y-polarized resonance exhibits

(a) a different strain dependence of the resonance wavelength, and **(b)** a drop in quality factor at small strain/θ, consistent with radiative coupling to the uncurved beams aligned with the y-axis. We note that the wavelength tuning range is larger for y-polarization (520-640 nm compared to 580-640 nm) and the maximum quality factor is also higher (350 compared to 230). However, this comes at the expense of having large background reflectance.

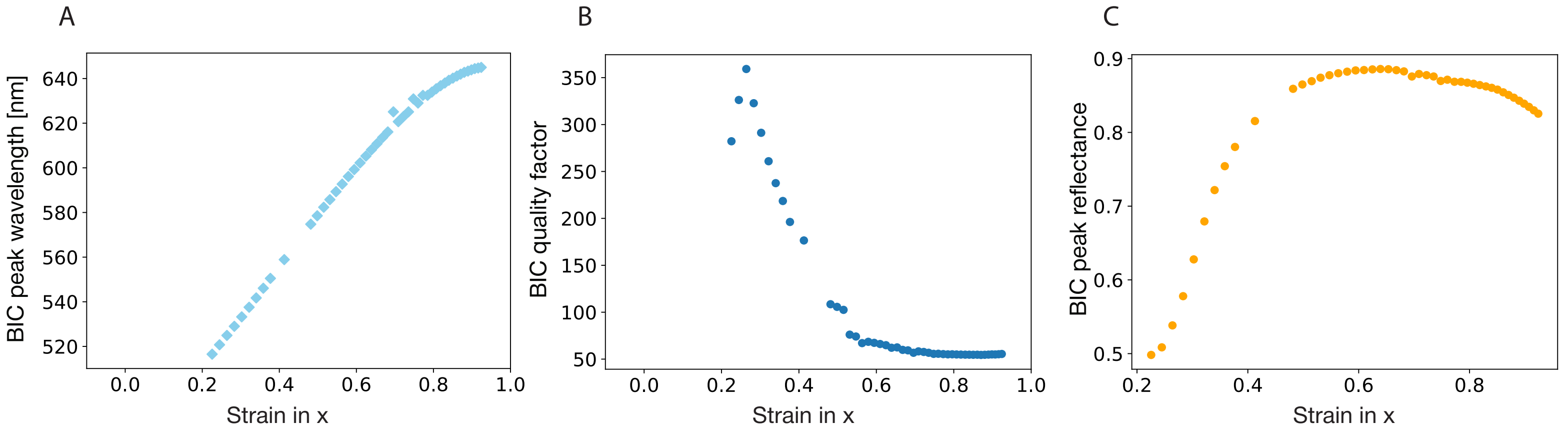

Figure S5: **Optical response of the beam-linked metasurface to y-polarized incident light.** Strain dependence of **(a)** the resonance wavelength, **(b)** the resonance quality factor, and **(c)** the resonance reflectance.

q-BIC dispersion

To corroborate the nonlocal nature of the resonant modes, we study the angle-dependent response and map out the associated band diagram. Figure S6 plots the resonance wavelength versus the in-plane momentum $k_{\parallel}/k_0 = \sin\phi$. The clear shift of the resonance wavelength (strong dispersion) with $k_{\parallel}$ shows that the mode is not just a local particle (Mie) resonance but instead a collective lattice mode that arises from the periodic array.

Figure S7 plots the inverse quality factor $1/Q$ versus $(k_{\parallel}/k_0)^2$. The nearly linear dependence is the hallmark of a bound state in the continuum (BIC): at the Γ-point ($\phi = 0$) the resonance is symmetry-protected, while away from Γ the radiative leakage increases quadratically with in-plane momentum.[4,5] The finite offset reflects the quasi-BIC condition introduced by symmetry breaking.

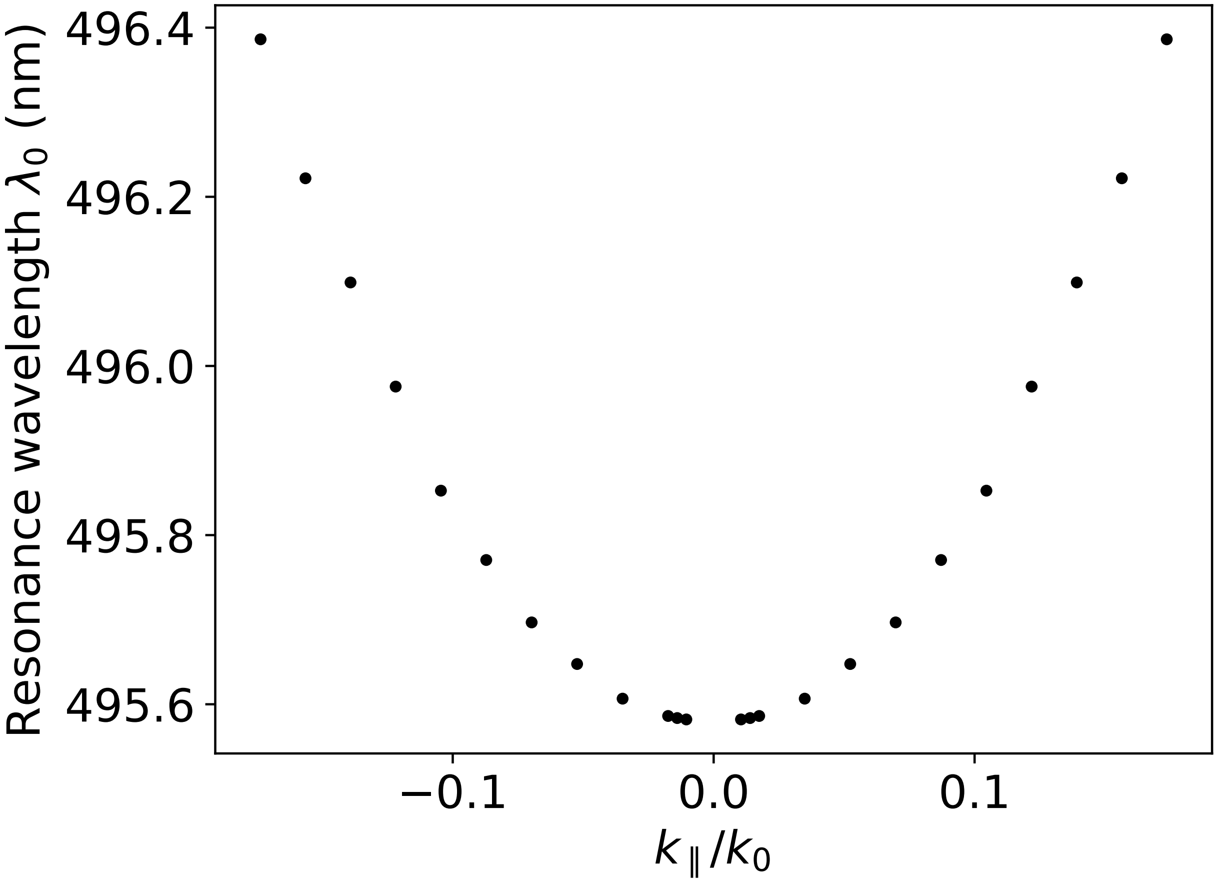

Figure S6: **Dispersion of the resonance nears the Γ-point.** The resonance wavelength λ_0 as a function of the normalized in-plane momentum $k_\parallel/k_0 = \sin(\phi)$, extracted from angle-resolved reflectance spectra. The clear angular dispersion of the resonance demonstrates its nonlocality, in contrast to a local Mie resonance which would be independent of incident angle.

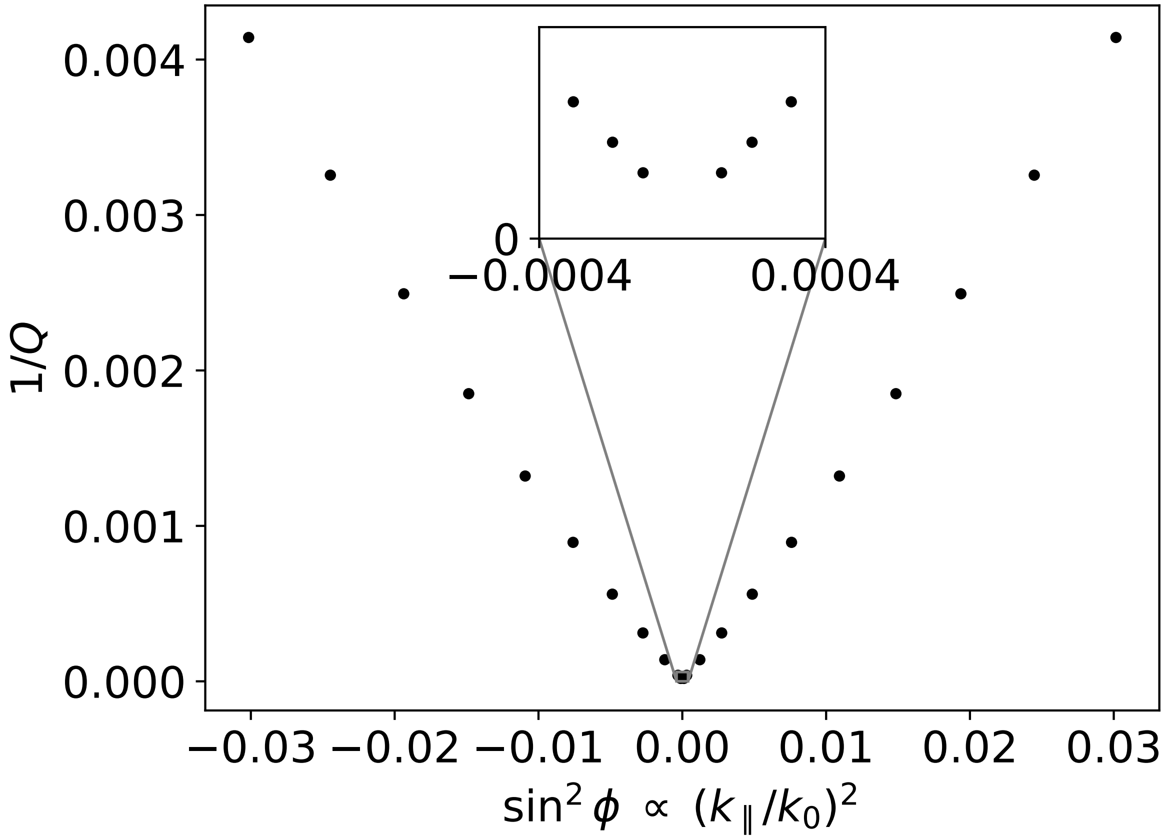

Figure S7: **Quadraticinverse quality factor scaling near the Γ-point.** The Inverse quality factor Q as a function of the normalized in-plane momentum squared $(k_{\|}/k_0)^2$. The linear dependence confirms the quadratic scaling of the radiative coupling characteristic of a symmetry-protected bound state in the continuum (BIC).

Actuation

To quantify the requirements for applying strain to the metasurface, we simulated the mechanical response of a finite array consisting of 20×20 unit cells with rigid handholds at the left and right edges. Strain is imposed by displacing one clamp while keeping the opposite side fixed. The total displacement needed for a target strain of 0.6 is 6 μm.

The simulated force-strain curve in Fig. S8 shows that the required force increases smoothly with strain, reaching $\sim 2\mu$N at $\epsilon = 0.6$. This places the required actuation well within the accessible range of MEMS actuators. Importantly, the array stiffness scales linearly with device size.

Considering the 50 nm height of the membrane, a simple parallel-plate capacitor to electrostatically actuate the device would require impractically high voltages ($\gg 5kV$). Therefore, we consider standard in-plane MEMS/micro mechanical actuators that can be fabricated next to the membrane and couple to the metasurface via rigid boundaries at its left/right edges (as in our finite-array simulations - *finite size effects* section in the main text). Several scalable options from literature seem compatible with a 50-nm monocrystalline silicon membrane such as electrostatic comb drives,[6] electrostatic zipper actuators,[7] piezoelectric stacks with flexure amplifiers,[8] thermal bimorph actuators.[9]

Out-of-plane stability

Kirigami sheets can exhibit out-of-plane (OOP) modes under in-plane (IP) loading.[10] In our geometry, OOP buckling is strongly disfavored by the beam cross-section and rigid "handhold" boundaries.[11]

A simple stiffness estimate rationalizes the suppression of OOP buckling in our geometry. For a ligament of width w (in-plane) and thickness t (out-of-plane), the bending second moments are

$$I_{\mathrm{OOP}} = \frac{wt^3}{12} \quad \text{(S7)}$$

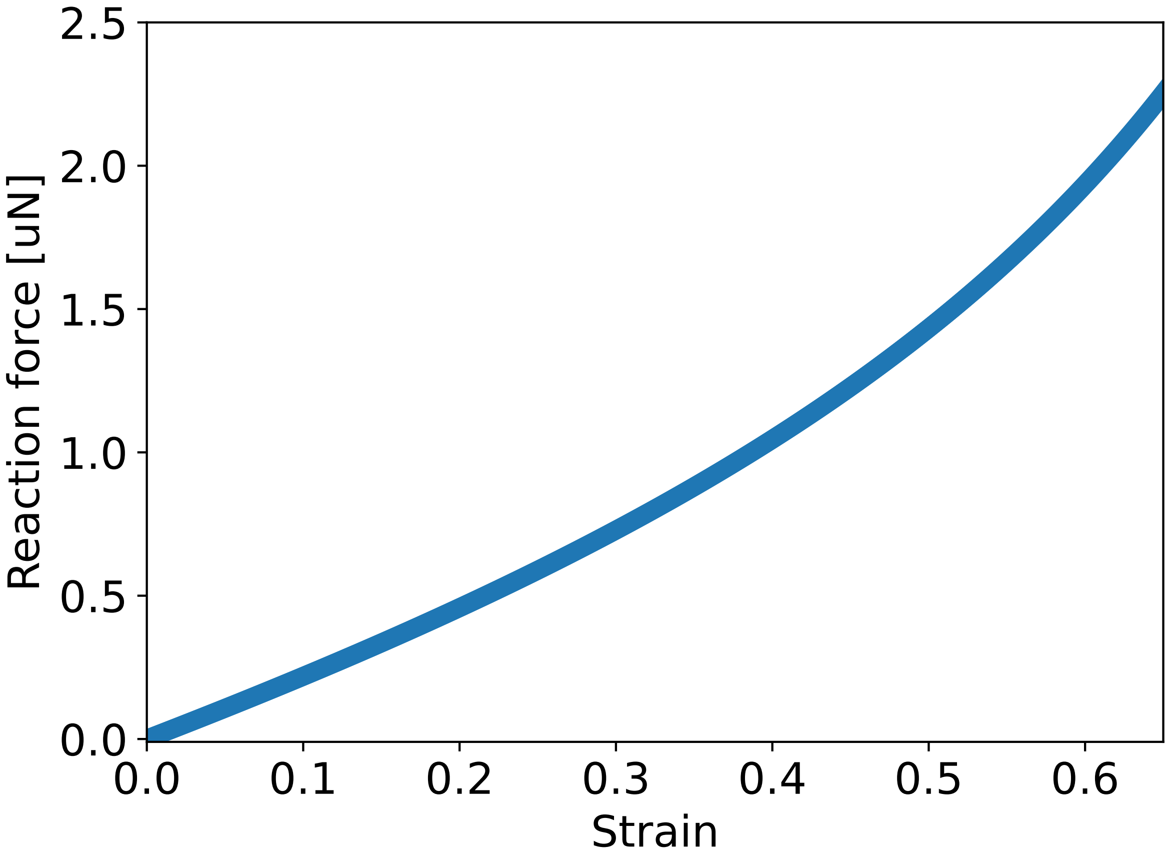

Figure S8: **Force-strain curve for a 20×20 array.** The total displacement for a strain of 0.6 for an array of this size is 6 μm. The total force required for this displacement for just the array is 2 μN.

and

$$I_{\mathrm{IP}} = \frac{tw^3}{12}, \tag{S8}$$

so their ratio $I_{\mathrm{OOP}}/I_{\mathrm{IP}} = (t/w)^2$. With $t = 50$ nm and $w = 10$ nm, $I_{\mathrm{OOP}} \gg I_{\mathrm{IP}}$, implying a substantially higher bending energy for OOP modes than for the intended in-plane mechanism. Practically, maintaining $t \gg w$ provides a large separation between OOP and IP modes. Using symmetric clamp motion further raises the OOP threshold without impeding the in-plane actuation.

Stress distribution

The stress distribution in the finite array correlates strongly with the local strain map in Fig.**5a,b**. However, as shown in figure S9, the edges and corners of the array experience roughly twice the stress as the center. A more optimized design - especially of the corners - can likely reduce this significantly. For example, we could remove one of the ellipsoidal particles at the corners to extend the beam.

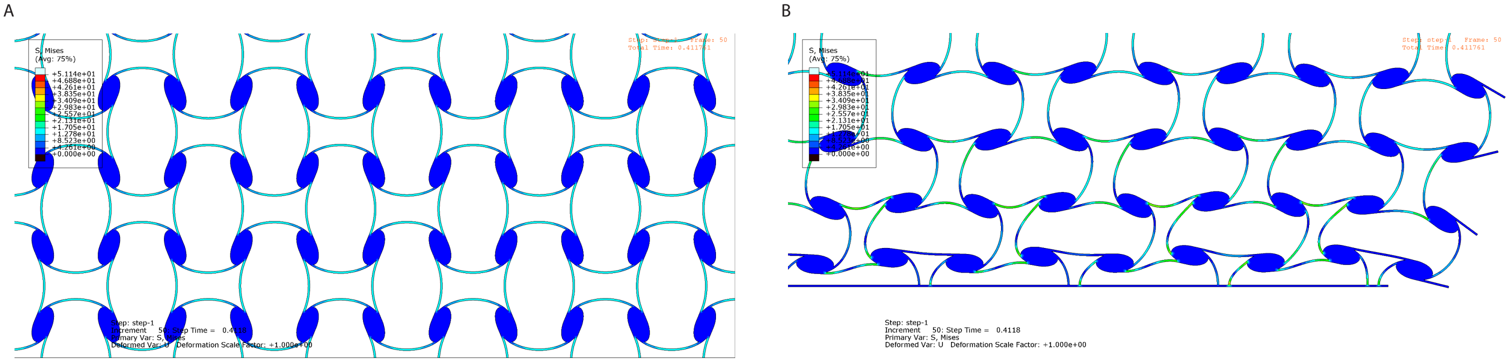

Figure S9: **Localized von Mises stress for a finite array with rigid boundaries on the left and right sides.** The stress for the center of the array **(a)** is about half of that at the edge of the array **(b)** for the operational strain range. At the edge the stress is more localized to a few connecting beams at the corners.

Fatigue life

In the main text we mention a fracture strength of 12-16 GPa for silicon nanobeams. The maximum simulated von Mises stress ($<$10 GPa at $\varepsilon = 0.6$) therefore corresponds to a safety factor of 1.2-1.6 relative to the reported fracture strength. Of course, fracture strength alone is not the full story. Silicon fracture is dominated by surface flaws rather than classical fatigue. Cyclic loading below 80% of fracture strength shows negligible degradation over $10^6 - 10^8$ cycles.[12] Our operational stresses fall within this safe regime for most of the structure. To maximize durability, standard nanofabrication practices, such as cryogenic etching, should be applied to mitigate side-wall roughness and stress-corrosion effects in

humid air.[13] Rounded beam junctions further reduce stress concentration factors, improving long-term reliability.

References

(1) COMSOL COMSOL Multiphysics Reference Manual. 2020.

(2) Schinke, C.; Christian Peest, P.; Schmidt, J.; Brendel, R.; Bothe, K.; Vogt, M. R.; Kröger, I.; Winter, S.; Schirmacher, A.; Lim, S.; Nguyen, H. T.; MacDonald, D. Uncertainty analysis for the coefficient of band-to-band absorption of crystalline silicon. *AIP Advances* **2015**, *5*, 067168.

(3) Smith, M. *ABAQUS/Standard User's Manual, Version 6.9*; Dassault Systèmes Simulia Corp: United States, 2009.

(4) Koshelev, K.; Lepeshov, S.; Liu, M.; Bogdanov, A.; Kivshar, Y. Asymmetric metasurfaces with high-Q resonances governed by bound states in the Continuum. *Phys. Rev. Lett.* **2018**, *121*, 193903.

(5) Hsu, C. W.; Zhen, B.; Stone, A. D.; Joannopoulos, J. D.; Soljačić, M. Bound states in the Continuum. *Nature Reviews Materials* **2016**, *1*, 16048.

(6) Judy, J. W. Microelectromechanical systems (MEMS): Fabrication, design and applications. *Smart Materials and Structures* **2001**, *10*, 1115–1134.

(7) Pelesko, J. A.; Bernstein, D. H. *Modeling MEMS and NEMS*; Chapman & Hall/CRC, 2014.

(8) Smith, S. T. *Flexures: Elements of elastic mechanisms*, 1st ed.; Taylor & Francis, 2000.

(9) Comtois, J. H.; Bright, V. M. Applications for surface-micromachined polysilicon thermal actuators and arrays. *Sensors and Actuators A: Physical* **1997**, *58*, 19–25, Micromechanics Sections of Sensors and Actuators.

(10) Dudek, K. K.; Kadic, M.; Coulais, C.; Bertoldi, K. Shape-morphing metamaterials. *Nature Reviews Materials* **2025**, *10*, 783–798.

(11) Rafsanjani, A.; Bertoldi, K. Buckling-induced Kirigami. *Phys. Rev. Lett.* **2017**, *118*, 084301.

(12) Muhlstein, C.; Brown, S.; Ritchie, R. High-cycle fatigue and durability of polycrystalline silicon thin films in ambient air. *Sensors and Actuators A: Physical* **2001**, *94*, 177–188.

(13) Gaither, M. S.; Gates, R. S.; Kirkpatrick, R.; Cook, R. F.; DelRio, F. W. Etching Process Effects on Surface Structure, Fracture Strength, and Reliability of Single-Crystal Silicon Theta-Like Specimens. *Journal of Microelectromechanical Systems* **2013**, *22*, 589–602.
